# Clinical Outcome and Toxicity in the Treatment of Anaplastic Thyroid Cancer in Elderly Patients

**DOI:** 10.3390/jcm9103231

**Published:** 2020-10-09

**Authors:** Teresa Augustin, Dmytro Oliinyk, Viktoria Florentine Koehler, Josefine Rauch, Claus Belka, Christine Spitzweg, Lukas Käsmann

**Affiliations:** 1 Department of Radiation Oncology, University Hospital, LMU Munich, 80539 Munich, Germany; teresa.augustin@med.uni-muenchen.de (T.A.); dmytro.oliinyk@med.uni-muenchen.de (D.O.); josefine.rauch@med.uni-muenchen.de (J.R.); claus.belka@med.uni-muenchen.de (C.B.); 2 Department of Internal Medicine IV, University Hospital, LMU Munich, 80539 Munich, Germany; viktoria.koehler@med.uni-muenchen.de (V.F.K.); christine.spitzweg@med.uni-muenchen.de (C.S.); 3 German Cancer Consortium (DKTK), Partner Site Munich, 81377 Munich, Germany

**Keywords:** ATC, anaplastic thyroid cancer, elderly, irradiation, survival

## Abstract

Background: The present study aims to evaluate the outcomes and toxicity of elderly anaplastic thyroid cancer (ATC) patients receiving (chemo)radiotherapy, as well as to identify prognostic factors. Patients and methods: A systematic review using the MEDLINE/PubMed and Cochrane databases was performed. Individual data from all eligible studies were extracted, and a pooled analysis (*n* = 186) was conducted to examine patient characteristics and treatment. All consecutive ATC patients (≥65 years) treated between 2009 and 2019 at our institution were evaluated for outcomes concerning progression-free survival (PFS), overall survival (OS) probabilities and treatment-related toxicity. Results: The systematic review and pooled analysis identified age as a prognostic factor. The median OS of our patient cohort (*n* = 26) was three months (range = 0–125). The 6-, 12- and 24-month survival rates were 35%, 22% and 11%, respectively. In the univariate analysis, a Karnofsky performance status of >70%, the Union for International Cancer Control Tumor–Node–Metastasis classification, multimodal therapy and an EQD2 of >49 Gy were correlated with longer OS and PFS. The acute grade 3 toxicity of dysphagia, dyspnea, dermatitis, mucositis and dysphonia was found in 23%, 15%, 12%, 12% and 8% of patients. Conclusion: Age appears to be a prognostic factor in ATC. Elderly ATC patients can tolerate multimodal treatment and achieve a promising outcome. Prospective studies need to confirm our findings.

## 1. Introduction

Anaplastic thyroid cancer (ATC) is one of the rarest, yet one of the most lethal, carcinomas that is seen in the human body. It only accounts for 1–2% [1,2,3,4] of all known thyroid carcinomas per year; however, it is responsible for about 50% of thyroid-cancer-associated deaths [2,4,5]. Its aggressive growth leads to the rapid infiltration of vital adjacent organs, such as the trachea, larynx and esophagus, as well as neck vessels, nerves and muscles. Additionally, early metastases commonly to lung and bones [6,7,8,9] result in fatal outcomes with a median overall survival (OS) that ranges between three and six months [10]. The overall one-year survival rate is only 10–20% [1,4,5,11,12,13,14].

The optimal treatment of ATC remains unknown. Due to its low incidence, large prospective trials are rarely performed. However, several studies propose a multimodal therapy regime, consisting of radical resection, radiotherapy and chemotherapy, to improve outcomes in ATC patients [3,11,13,14,15]. Despite this, survival has remained relatively stable over the past decades [2], especially in elderly patients, who make up an important subgroup of ATC patients, among which prognosis is very poor [3,4,12,15,16,17,18]. This group of people often represents a combination of several comorbidities, immunodeficiencies and organ dysfunctions and may not tolerate aggressive treatment [19,20]. In contrast, elderly patients with a poor prognosis should spend as little of their remaining lifetime attending oncologic treatments and are, therefore, better candidates for short treatments including hypofractionated radiotherapy [21]. These considerations mean that it is important to judge a patient’s survival time as accurately as possible to personalize treatment approaches.

We aim to perform a systematic review using the MEDLINE/PubMed and Cochrane databases to evaluate patients’ age as a prognostic factor. Individual data from all eligible studies will be extracted and pooled in order to examine patient characteristics and treatment. Furthermore, all consecutive ATC patients ≥ 65 years at initial diagnosis will be investigated concerning outcome and treatment-related toxicity and prognostic factors of OS and progression-free survival (PFS) will be identified.

## 2. Patients and Methods

### 2.1. Systematic Review of Literature

A systematic review of the literature was undertaken using PubMed/MEDLINE and Cochrane databases following the Preferred Reporting Items for Systematic Reviews and Meta-Analyses (PRISMA) protocol. Abstracts were screened for eligibility so that the most important articles were analyzed by full-text screening. Inclusion criteria were based on the study setting. Age was investigated as one of the prognostic factors in the uni-/multivariate analyses. Furthermore, treatment specifications and cut-off values for age of included studies were analyzed. Reviews, case reports, experimental data, personalized treatments, drug trials or publications arising conflict of interests were excluded.

### 2.2. Pooled Analysis

Eligible publications providing raw data on age, TNM/UICC stage distribution, treatment (e.g., surgery, radiotherapy, chemotherapy) and outcome were extracted and evaluated in order to examine patient- and treatment-related characteristics as well as the outcomes of ATC patients.

Statistical analyses were performed using SPSS statistics 25 (IBM, Chicago, IL, USA). Subgroups were compared using the log-rank test. For all statistical analyses, a significance level of α = 0.05 was defined.

### 2.3. Single-Center Patient Cohort

The retrospective study included data from 26 consecutive patients diagnosed with ATC between 2009 and 2019 at our center. The study protocol was approved by the ethics committee of the Ludwig Maximilian University of Munich (Munich, Germany) (Approval Number: 19–885).

### 2.4. Data Acquisition

Data were analyzed according to ten patient- and treatment-related characteristics: age, gender, Karnofsky performance status (KPS), the Union for International Cancer Control classification (UICC stage), nodal involvement, distant metastases, radiation technique, performance of surgery, chemotherapy and radiation dose escalation. Inclusion criteria were patients ≥ 65 years with a histologically confirmed ATC, staged according to the revised 8th edition of the Union for International Cancer Control Tumor–Node–Metastasis (UICC TNM) classification. The information was gained from pathological reports, which were available in all 26 cases. The study endpoints were the 6-, 12- and 24-month OS and PFS. Multimodal treatment was defined based on earlier reports such as trimodal therapy containing surgical resection and postoperative chemoradiotherapy (CRT) [22].

### 2.5. Criteria for Multimodal Treatment Approach

In accordance with the guidelines of the European Society for Medical Oncology (ESMO), surgical resection of the tumor burden (partial or total) was only performed in patients with a prospect of achieving R0/R1 status and was based on the perioperative risk assessment, as well as on comorbidities [11]. Importantly, M0 status was not an exclusion criterion for surgery. Similarly, radical CRT was performed subsequently if no absolute contraindications arose, such as a KPS status of <40% and/or poor liver or kidney function and cardiovascular comorbidities. Relative contraindications were discussed within multidisciplinary tumor boards consisting of surgeons, radiation oncologists and oncologists.

### 2.6. Statistical Analysis

Statistical analyses were performed using SPSS Statistics 25 (IBM, Chicago, IL, USA). Subgroups were compared by a log-rank test. All significant variables in the univariate analysis were included in a multivariate Cox regression analysis. The proportional hazard assumption of the Cox regression analysis was tested. PFS was defined as the time between the last day of radiotherapy and the occurrence of local or distant progression or death from all causes. OS was defined as the time between the last day of radiotherapy and death. For all statistical analyses, *p* ≤ 0.05 was considered statistically significant.

## 3. Results

### 3.1. Systematic Review of Literature

**Figure 1:**
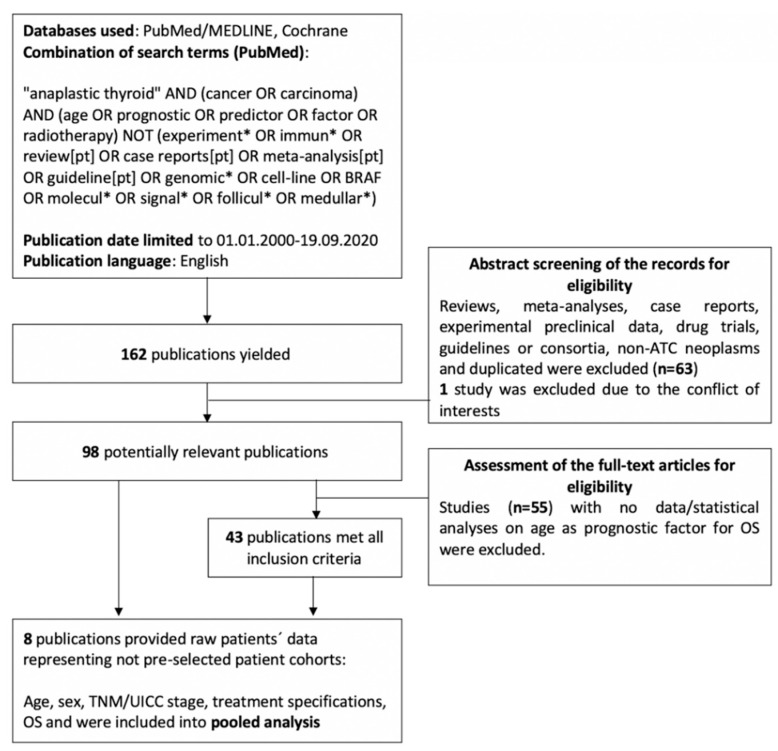
PRISMA flowchart for the systematic review.

Our search criteria with combinations of terms and operators are shown in Figure 1. In total, 162 publications were yielded using PubMed/MEDLINE databases. The Cochrane database did not provide any additional studies. Abstracts of these studies were screened for eligibility and excluded for the reasons shown in Figure 1. Ninety-eight potentially relevant publications underwent full-text assessment for eligibility and are included in Table 1. The matching criteria are shown in Figure 1. As a result, 43 publications were included in our systematic review of the literature with a total of 15 722 ATC patients diagnosed or analyzed in the past 20 years. In 33 (76.7%) of the included studies, younger age was significantly associated with a favorable outcome, at least in the univariate analysis. Furthermore, in 23 (53.5%) publications, age achieved significance in the multivariate analysis. Importantly, the most commonly chosen cut-off values for age were 70 (21%) and 65 (18.6%) years, representing cohorts of 2213 and 7923 patients, respectively. A review of the literature was undertaken by two authors (T.A. and D.O.) in order to minimize the risk of selection bias.

### 3.2. Results of the Pooled Analysis

The individual patient data of eight eligible publications were extracted (*n* = 186) (Table 2) [49,56,57,58,59,60,61,62]. The median age at initial diagnosis was 68 (range = 35–92) years. Treatment consisted of surgery in 95 (51%), radiotherapy in 152 (82%) and sequential or concurrent chemotherapy in 114 (61%) of all patients. Multimodal treatment containing surgery followed by postoperative chemoradiotherapy was administered in 74 (40%) patients. Fifty-one (27%) patients were diagnosed with metastatic disease (UICC stage IVC).

The median OS was 5.9 months (range: 0–157). Survival rates at 6, 12 and 24 months were 50%, 24% and 15%, respectively. Surgery (*p* < 0.001), radiotherapy (*p* < 0.001), sequential or concurrent chemotherapy (*p* < 0.001) and administering multimodal treatment (*p* < 0.001) were prognostic factors concerning OS in the univariate analysis. In the multivariate analysis, radiotherapy (*p* < 0.001, hazard ratio (HR) = 0.383, 95% confidence interval (CI) = 0.253–0.579) was significantly associated with an improved OS, whereas surgery (*p* = 0.107, HR = 0.640, 95% CI = 0.372–1.100), sequential or concurrent chemotherapy (*p* = 0.067, HR = 0.664, 95% CI = 0.428–1.029) and multimodal treatment (*p* = 0.464, HR = 0.777, 95% CI = 0.396–1.526) did not achieve significance in the multivariate analysis.

### 3.3. Propensity Score Matching (PSM)

**Figure 2:**
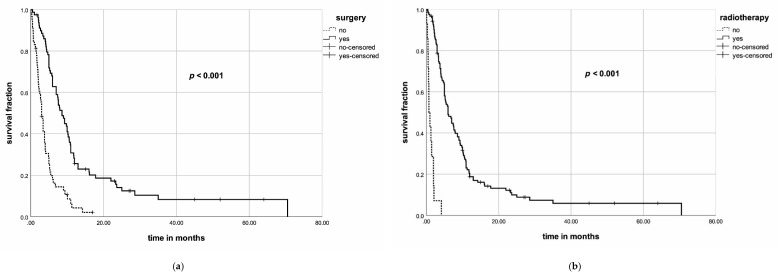

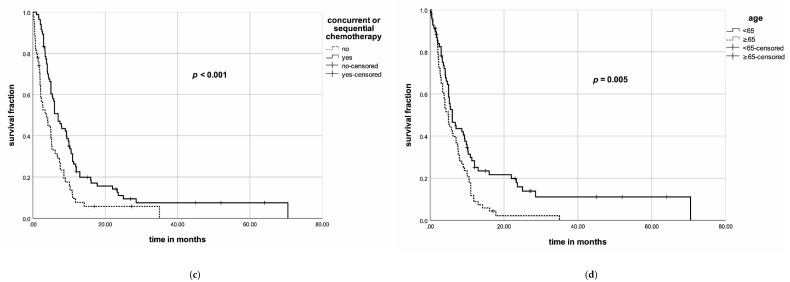
(**a**). Kaplan–Meier curve for surgery and overall survival in the univariate propensity score matching (PSM) analysis; (**b**) Kaplan–Meier curve for radiotherapy and overall survival in the univariate PSM analysis; (**c**) Kaplan–Meier curve for sequential or concurrent chemotherapy and overall survival in the univariate PSM analysis; (**d**) Kaplan–Meier curve for age and overall survival in the univariate PSM analysis.

Individual patients’ data of all eligible patient cohorts [49,56,57,58,59,60,61,62] were included according to our database assessment protocol in the propensity score matching (PSM) analysis. Patients aged <64 years were matched in a 1:1 ratio to patients aged ≥65 years. To each patient aged <64 years, one corresponding patient aged ≥65 years with exactly the same UICC stage (IVA/B vs. IVC) was matched. PSM also considered the treatment mode, including surgery and chemotherapy. Sixty-nine patients aged <64 years were matched to 69 patients aged ≥65 years (Table 3). Surgery (*p* < 0.001), radiotherapy (*p* < 0.001), concurrent or sequential chemotherapy (*p* < 0.001) and younger age (*p* = 0.005) were associated with an improved OS in the univariate analysis (Figure 2), whereas gender did not achieve significance (*p* = 0.96). In the multivariate analysis, surgery (*p* < 0.001, HR = 0.294, 95% CI = 0.192–0.45), radiotherapy (*p* < 0.001, HR = 0.042, 95% CI = 0.018–0.098) and younger age (*p* = 0.008, HR = 1.721, 95% CI = 1.151–2.573) were significantly associated with an improved OS, whereas concurrent or sequential chemotherapy (*p* = 0.171, HR = 1.406, 95% CI = 0.863–2.289) failed to achieve significance.

### 3.4. Patient Characteristics of Our Single-Center Cohort

The median age at initial diagnosis was 74 (65–97) years and 13 (50%) of all patients were female. The Karnofsky performance status (KPS) was ≤70% in 12 (46%) and >70% in 14 patients (54%). In only one (4%) patient, the disease was limited to the thyroid gland (stage IVA). Nine (35%) patients had extrathyroidal infiltrations (stage IVB) and 16 (62%) already showed distant metastases (stage IVC), respectively (Table 4). At initial diagnosis, 62% of patients had distant metastases that were found in one (44%), two (44%), three (6%) or four (6%) different organs. Ninety-four percent of the metastases were localized pulmonary, 50% lymphatic, 19% osseous, 6% hepatic and 6% cerebral (Table 2). Twelve patients (46%) were treated in a multimodal approach (Table 5 + CRT cohort).

### 3.5. Treatment-Related Characteristics

A hemithyroidectomy was performed in four (15%) patients, total and subtotal thyroidectomy in six (23%) and two (8%) patients, respectively. Chemotherapy was administered in 13 (50%) patients. Of those, six (46%) patients received concurrent chemotherapy with carboplatin (area under the curve (AUC) = 2) and administered 50 mg/m^2^ paclitaxel weekly. Single-agent chemotherapy with doxorubicin (10 or 20 mg/m^2^) was given weekly concurrent to radiation in five (38%) patients. Sequential chemotherapy was given in two (15%) patients (paclitaxel with carboplatin or pemetrexed). Irradiation was administered using a three-dimensional conformal radiotherapy (3D-CRT) technique in 17 (65%) patients. Nine (35%) patients were treated using intensity-modulated radiation therapy (IMRT) (Table 5). The cumulative radiation dose was calculated in equivalent dose in 2 Gy fractions (EQD2). The median EQD2 was 49 Gy (range = 5–71).

### 3.6. Treatment-Related Toxicities

Treatment-emerged adverse events (TEAE) were evaluated according to the Common Terminology Criteria for Adverse Events (CTCAE) Version 4. The most common side effects were dysphagia, dermatitis, mucositis, dyspnea and dysphonia. Acute grade 3 toxicity of dysphagia, dyspnea, dermatitis, mucositis and dysphonia was found in 23%, 15%, 12%, 12% and 8% of patients. Therapy-related toxicity grade 4/5 was not observed. An EQD2 of ≥40 Gy was associated with radiation-induced dermatitis grade ≥ 2 (*p* = 0.04), as well as with dysphagia grade ≥ 2 (*p* = 0.005) and mucositis grade ≥ 2 (*p* = 0.04). Dyspnea grade ≥ 2 was not correlated with an EQD2 of ≥40 Gy (*p* = 0.07).

### 3.7. Outcomes on Survival and Relapse in the Single-Center Evaluation

The median OS after the end of radiotherapy was three months (range = 0–125, 95% confidence interval (CI) = 0.75–5.29). The 6-, 12- and 24-month survival rates were 35%, 22% and 11%, respectively. The median PFS after the end of radiotherapy was two months (range = 0–125, 95% CI = 0.34–3.66). Local recurrence was observed in three (12%) patients during follow-up.

### 3.8. Patient- and Treatment-Related Factors of Prognosis in the Single-Center Evaluation

**Figure 3:**
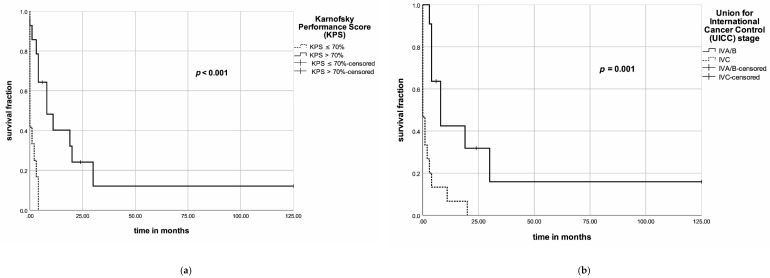

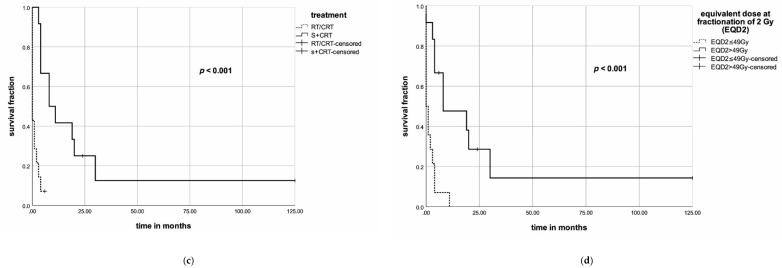
(**a**) Kaplan–Meier diagram for the Karnofsky performance status (KPS) and overall survival; (**b**) Kaplan–Meier diagram for the UICC stage and overall survival; (**c**) Kaplan–Meier diagram for treatment approaches and overall survival; (**d**) Kaplan–Meier diagram for EDQ2 levels and overall survival.

**Figure 4:**
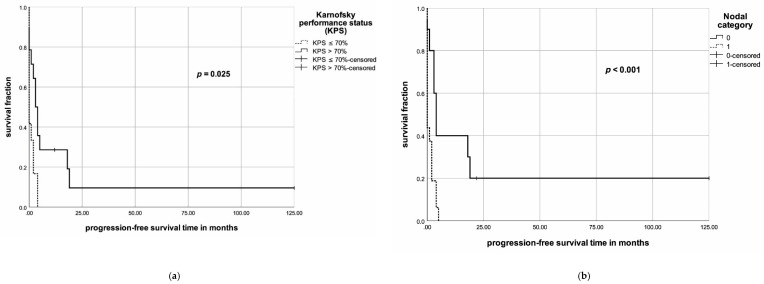

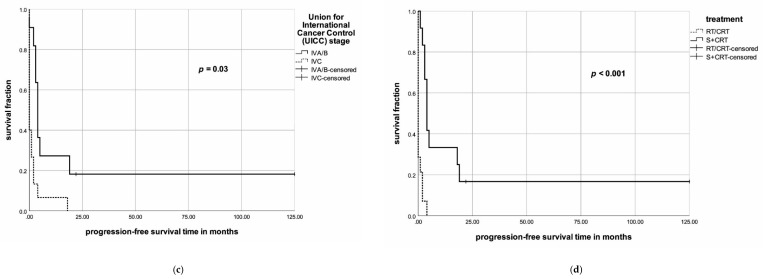

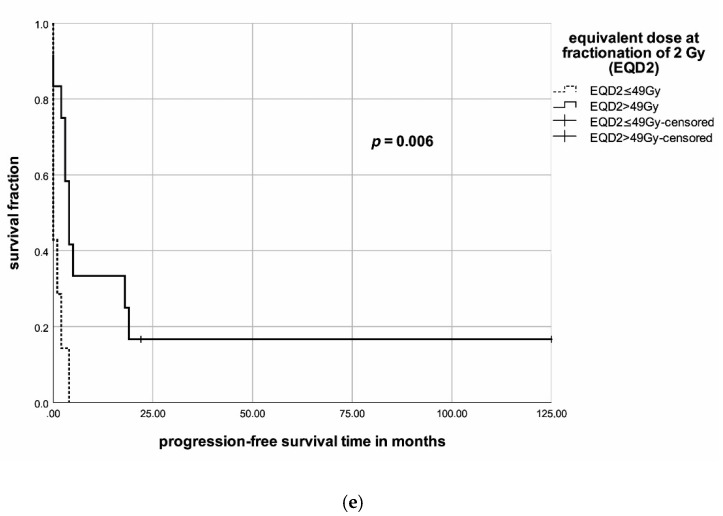
(**a**) Kaplan–Meier diagram for the KPS and progression-free survival (PFS); (**b**) Kaplan–Meier diagram for N status and PFS; (**c**) Kaplan–Meier diagram for the UICC stage and PFS; (**d**) Kaplan–Meier diagram for treatment approaches and PFS; (**e**) Kaplan–Meier diagram for EDQ2 levels and PFS.

In the univariate analyses, KPS (>70%), N category, M category, UICC stage, surgery, multimodal treatment and an EQD2 of >49Gy were associated with an improved OS (Figure 3). In the multivariate analysis, for OS, none of the following factors achieved significance: KPS (hazard ratio (HR) = 0.42, 95% confidence interval (95% CI) = 0.07–2.64, *p* = 0.36), UICC stage (HR = 1.45, 95% CI = 0.34–6.22, *p* = 0.62), multimodal therapy (HR = 0.52, 95% CI = 0.07–3.91, *p* = 0.52) or EQD2 level (HR = 0.56, 95% CI = 0.14–2.32, *p* = 0.43) (Table 6). Univariate analysis of PFS, KPS (>70%), N category, M category, surgery, multimodal therapy and an EQD2 > 49 Gy resulted in improved PFS (Figure 4a–e). In the multivariate analysis, none of the following factors had a significant impact on PFS (Table 7): KPS (HR = 1.63, 95% CI = 0.35–7.45, *p* = 0.53), N category (HR = 1.81, 95% CI = 0.56–5.92, *p* = 0.33), M category (HR = 1.94, 95% CI = 0.45–8.32, *p* = 0.37), multimodal therapy (HR = 0.41, 95% CI = 0.06–2.72, *p* = 0.35) or EQD2 level (HR = 0.95, 95% CI = 0.21–4.34, *p* = 0.94).

## 4. Discussion

The main goal of this report was to investigate the prognostic impact of age in the treatment of ATC, as well as to study real-world clinical data and outcomes from elderly patients with ATC who received multimodal therapy outside the framework of a clinical trial. To our knowledge, this is the first comprehensive experience reported to date, evaluating patients aged ≥65 years in order to investigate the outcomes concerning OS and PFS, treatment-related toxicity and prognostic factors.

In general, age appears to be an important risk factor for the outcomes in patients with ATC [3,4,12,15,16,17,18]. Two multicenter studies with almost 3000 patients found increasing age as a prognostic factor, resulting in a less favorable outcome [3,17]. In the study of Wendler et al. with 100 patients, an age > 70 was found to be an independent prognostic factor for shorter OS [16]. This is in accordance with a large registry study from Japan that included 677 ATC patients [4]. They also found an age > 70 associated with a decreased OS, while an analysis of Surveillance, Epidemiology and End Results Program (SEER) data with 516 patients reports that patients older than 60 years already suffer from higher mortality rates [18]. Their data show a difference of 28% in cancer-specific survival (CSS) after a follow-up of one year when comparing patients over 60 with those under 60 years of age. On the other hand, the single-center cohort with 54 patients from Rao et al. found no association of patients above 60 years with worse OS (*p* = 0.5). This might be due to the small cohort and a relatively low median age of 63 years [14].

Wendler et al. confirmed that age has a severe impact on treatment allocation. In patients <60 years, 77% received multimodal therapy, while in the group >80 years only 17% received this aggressive treatment approach [16]. Unfortunately, no reasons are given here for the individual assignments of therapies or conclusions regarding quality of life.

Based on the results of our systematic review and pooled analysis, age appears to have a prognostic impact on the outcome concerning OS. Elderly patients (aged ≥65 years) showed a significant association with poorer OS compared to younger patients. Therefore, elderly patients need to be considered as a special patient group in ATC treatment.

The KPS represents an important prognostic factor for OS and PFS in several types of cancer [20,63,64,65]. In our cohort, all patients with a KPS ≤ 70% died in less than six months. On the other hand, for patients with a KPS > 70%, the 6-, 12- and 24-month survival rates were 64%, 40% and even 20%, respectively. In ATC, KPS, as well as the Eastern Cooperative Oncology Group Performance Index (ECOG), are not frequently reported in the literature and their prognostic value remains controversial. Future studies need to address this issue and provide a performance status, e.g., ECOG or KPS, in order to consequently prevent selection bias.

Nodal involvement and distant metastases determine the UICC stage and are, therefore, important for clinical outcomes. According to Wendler et al. and Glaser et al. [16,43], nodal involvement impacts OS negatively. Additionally, many larger and smaller studies report that patients with distant metastases experience a dismal prognosis [4,5,6,12,16,43,50]. In our study cohort, local nodal involvement and distant metastases were associated with poor outcome, which corresponds with the published literature. We found a six-month overall survival rate of patients with nodal involvement at an initial diagnosis of 25%, while it was 50% for those who did not have nodal involvement at that time.

The UICC stage represents a clinically important prognostic factor for OS. In our study, patients were diagnosed according to the revised eighth edition of the UICC TNM classification. We found that OS, as well as PFS, strongly depend on the stage. The 6- and 12-month survival rates were as follows: 100% each in IVA stage; 67% and 40%, respectively, for stage IVB; and 13% and 6%, respectively, for stage IVC. Similarly, the results from the studies by Haymart et al. and Wendler et al. are consistent with our findings [3,16].

Importantly, more than 40% of all ATC cases occur in advanced stages, which means that symptoms of local compression with dyspnea and dysphagia and/or distant metastases are present [1,5,8,13,14,66]. These cases correspond to the unresectable stage IVB or stage IVC, in which, usually, no surgery or only an incomplete resection (R2) is possible [2,5,7]. In this situation, definitive chemoradiotherapy may provide local control and symptomatic relief [6,7,8,50].

According to the published literature, the administered radiation dose depends on treatment goals (palliative vs. curative treatment) and ranges mainly between 20 and 75 Gy [5,11]. Nevertheless, the exact radiation dose in curative settings remains highly controversial. We found a radiation dose of >49 Gy as a significant prognostic factor for OS and PFS, while other researchers described a dose of >60 Gy [5,22]. According to Fan et al., radiation doses of >60 Gy are associated with an improved local disease control (*p* < 0.001) and overall survival (*p* = 0.004). Differences were also found in the median OS for patients with radiotherapy (RT) doses of >60 Gy (10.6 months) vs. doses <60 Gy (3.6 months) [22]. Furthermore, the results of Glaser et al. show a more favorable outcome with higher-dose radiation (≥59.4 Gy) [43].

In accordance with the recent analysis of 1288 patients from the National Cancer Database (NCDB), radiotherapy can stop or delay the local growth process. As a result, patients with advancedstage IVB and IVC and unresectable tumors may benefit from more aggressive treatments. They found that patients who received radiation from 60 to 75 Gy had significantly better OS rates compared to patients with radiation doses from 45 to 59.9 Gy [5]. Our study found that a radiation dose of >49 Gy results in a more favorable OS, in addition to patients aged ≥65 years [6,11,12,16]. On the other hand, we found that an EQD2 of ≥40 Gy is associated with radiation dermatitis grade ≥2 (*p* = 0.04), as well as with dysphagia grade ≥2 (*p* = 0.005) and mucositis grade ≥2 (*p* = 0.04). Interestingly, dyspnea (*p* = 0.07) was not associated with an irradiation dose. According to Fan et al., irradiation with >60 Gy in patients resulted in no grade 4 subacute or later adverse effects. However, common acute grade 3 adverse events were reported for dermatitis (20%), mucositis (13%), dysphagia (8%) and fatigue (7%) [22]. Similarly, to the results of Fan et al., no treatment-related toxicity grade 4/5 was observed in our study cohort. In contrast, our study cohort showed acute grade 3 toxicity of dermatitis and mucositis both only in 12% of all patients, which might be due to lower radiation doses. Severe dysphagia, however, was present in 23% of our patients. The reasons and possible confounders for this relatively high percentage are potentially due to the close surveillance of our patients and the proactive insertion of a percutaneous endoscopic gastrostomy (PEG) at our center.

The implementation of new radiation delivery techniques such as Intensity-Modulated Radiotherapy (IMRT) achieved improved outcomes concerning OS and PFS with less toxicity compared to older radiation techniques like 2D/3D-CRT [48]. The study by Park et al., which included 41 patients, found that IMRT (*n* = 28) resulted in a more favorable OS (HR = 0.40, *p* = 0.005) and PFS (HR = 0.33, *p* = 0.005) compared to 3D-CRT (*n* = 13). In addition, higher radiation doses could be safely achieved using IMRT rather than 3D-CRT (median doses of 66 Gy vs. 60 Gy, *p* = 0.005) [48]. A small cohort study by He et al. confirmed that with IMRT, the dose tolerance was significantly improved; almost all patients received higher-dose radiation (>54 Gy) [67]. On the other hand, Corrigan et al. emphasized the recommendation of IMRT in the treatment of neck and head cancer, but little evidence was available regarding the treatment of ATC. However, they also found an association between IMRT and higher 12-month survival rates compared to 2/3D-CRT [15]. In our study, we found a benefit for IMRT at the 12-month survival rate compared to 3D-CRT (44% vs. 12% at 12 months). However, the difference was not significant.

The administration of chemotherapy in our cohort resulted in no further improvement of OS and PFS. Several studies confirm our controversial findings [12,15,50]. In contrast, two German studies found a survival benefit for administering concurrent or sequential chemotherapy to radiotherapy [16,44]. However, administering concurrent chemotherapy to radiotherapy in ATC remains highly controversial especially in elderly patients. Tiedje et al. recently summarized the latest evidence and confirmed that it is still unclear whether chemotherapy or chemoradiotherapy may improve patients’ outcomes. Moreover, administering chemotherapy only in stage IVC or also in stage IVA or IVB remains arguable [68].

Recent studies show that trimodal treatment (surgery, radiotherapy and chemotherapy) combined as a multimodal therapy significantly improves both OS and PFS in patients with ATC [3,5,11,13,14,15,16]. As a result, this multimodal therapy regime is increasingly becoming the standard of care, especially for patients in stage IVA and resectable stage IVB [1,12,14] and was incorporated into national and international guideline recommendations [1,69].

We found that elderly patients (≥65 years) appear to benefit from multimodal treatment including surgical resection followed by CRT compared to definitive chemo-/radiotherapy alone. Nonetheless, it failed to achieve significance in the multivariate analysis given the limitations of our study, such as limited patient number and the retrospective study design. The combination of surgery and chemoradiotherapy showed 6-, 12- and 24-month OS rates of 67%, 42% and 21%, respectively, compared to definitive chemo-/radiotherapy with 7%, 0% and 0%. Significantly improved PFS rates were also observed in 50%, 33% and 33% of patients compared to those with only definitive chemo-/radiotherapy of, again, 7%, 0% and 0%. Fan et al. observed in a cohort of 104 patients a 12-month OS rate of 54.7% in 53 patients who were treated with multimodal therapy. On the other hand, the 12-month overall survival rate in the 51 patients who were treated with concurrent chemoradiation or radiotherapy alone was only 12.8%. In the multivariate analysis, they also found multimodal treatment associated with improved local progression-free survival (LPFS) (*p* = 0.017). The 12-month LPFS rate in patients who were treated multimodally was 85.9% vs. 54.1% in those patients who were not (*p* = 0.003) [22]. Importantly, not all patients may tolerate combined or multimodal treatment approaches. Elderly patients with ATC need more attention and personalized treatment. In order to optimize such personalized approaches, the patients’ survival prognoses must be considered for decision-making. Therefore, our study revealed several prognostic factors, namely KPS, UICC, multimodal treatment and radiation dose escalation as well as outcome and toxicity in elderly patients.

Several limitations must be considered interpreting the results of the present study such as the retrospective nature and, therefore, a risk of including hidden selection and confounding biases. In addition, the patient cohort is relatively small with a long recruitment period.

According to our findings, treatment-related toxicity appears to be manageable in patients aged ≥65 years. Outcomes in elderly patients can be improved by more intensive therapy regimes such as combined treatments or dose escalation. We state that age does not need to be an exclusion factor for multimodal treatments and should be discussed within multidisciplinary tumor boards consisting of surgeons, oncologists and radiation oncologists.

## 5. Conclusions

Age is an independent prognostic factor in the treatment of ATC. Multimodal treatment including surgery and chemoradiotherapy in elderly patients with ATC appears to be associated with promising outcomes with manageable toxicity. Several prognostic factors for elderly patients were identified and may help physicians to estimate a patient’s prognosis and tailoring personalized treatment approaches. Despite the rare occurrence, ATC remains highly lethal, and therefore, prospective studies in elderly patients are needed in order to improve future outcomes.

## Figures and Tables

**Table 1:** Systematic review of the literature: age as a prognostic factor in patients with anaplastic thyroid cancer (ATC).

Author	Number of Patients (*N*)	Treatment	Age Cut-Off (Years)	Results
Sugitani et al. (2001) [23]	47	Multimodal—20% Other—80%	40–49 (7%) 50–59 (16%) 60–69 (34%) 70–79 (35%) 80–89 (7%)	Age was not a significant prognostic factor in the uni- or multivariate analysis
Pierie et al. (2002) [24]	67	Surgery—67% EBRT—84% ChT—31%	Cut-off: 70 ≤70 (45%) >70 (55%)	An age of ≤70 years was an independent predictor for beneficial OS (HR = 0.47, *p* < 0.023)
Kihara et al. (2004) [25]	19	Surgery—53% RT—68% ChT—63%	Cut-off: 70 40–49 (5%) 50–59 (0%) 60–69 (26%) 70–79 (32%) 80–89 (37%)	Age was not a significant prognostic factor in the uni- or multivariate analysis
Kebebew et al. (2005) [18]	516	Surgery—49% EBRT—63.2% ChT—not reported	Cut-off: 60 Mean: 71.3 (15–95)	An age of <60 years was an independent predictor for beneficial survival (HR = 0.482, 95% CI = 0.268–0.867, *p* < 0.05)
Brignardello et al. (2007) [26]	27	Surgery + adjuvant RT/ChT—56% Surgery + neoadjuvant RT/ChT—19% ChT alone—19%	Median: 70 (46–92)	Age was not a significant prognostic factor in the uni- or multivariate analysis
Kim et al. (2007) [27]	121	Unilateral palliative surgery—12% postoperative: 42.9% only RT, 7.1% only ChT and 14.3% both Bilateral curative surgery—59% postoperative: 50.7% only RT, 8.5% only ChT and 12.7% both RT alone—10.7% ChT alone—1.7% ChT/RT—4.1%	Cut-off: 60 <60 (33%) ≥60 (67%)	An age of <60 years was an independent predictor for lower disease-specific mortality(HR = 0.47, 95% CI = 0.30–0.74, *p =* 0.001)
Chen et al. (2008) [28]	261	Surgery only—26.1% EBRT alone—14.2% Surgery + EBRT—49.4%	<45 (5.7%) 45–54 (9.2%) 55–64 (19.9%) 65–74 (29.1%) 75–84 (23.4%) ≥85 (12.6%)	Younger age was an independent predictor for improved overall survival (HR = 1.02, 95% CI = 1.00–1.03, *p =* 0.007)
Yau et al. (2008) [29]	50	Surgery—68% EBRT—46% ChT—36%	Cut-off: 65 ≤65 (28%) >65 (72%)	In the univariate analysis, an age of ≤65 years was significantly associated with improved survival (*p =* 0.025) No significance in the multivariate analysis
Bhatia et al. (2009) [30]	53	Surgery—58.5% RT—100% CRT—73.6% Sequential ChT—16.9%	Median: 66.1 (27–88)	Age was not a significant prognostic factor in the uni- or multivariate analysis
Roche et al. (2010) [31]	26	Surgery—84.6% RT—53.8% ChT—19.2%	Mean: 75 (52.3–90.8)	Age >75 years was an independent predictor for poor prognosis (*p* < 0.05)
Akaishi et al. (2011) [32]	100	Surgery—70% RT—78% ChT—28%	Cut-off: 70 <70 (52%) ≥70 (48%)	Age ≥70 years was a significant risk factor for poorer survival in the multivariate analysis (RR = 1.03, 95% CI = 1.01–1.05, *p* = 0.014)
Derbel et al. (2011) [33]	44	Surgery alone—4.5% Surgery + CT—7% Surgery + RT + CT—79.5% RT alone—4.5% Surgery + RT—4.5%	Cut-off: 65 Median 65 (44–80)	An age of >65 years was associated with poorer outcome in the univariate analysis (HR = 2.36, 95% CI = 1.15–4.84, no *p*-value reported)
Sherman et al. (2011) [34]	37	Surgery + CRT—51% CRT—100%	Cut-off: 70 <70 (73%) ≥70 (27%)	An age of <70 years was an independent predictor for beneficial OS (HR = 0.32, 95% CI = 0.13–0.78, *p* = 0.013)
Tashima et al. (2011) [35]	33	Surgery—58% RT—52% ChT—39% RT + ChT—36%	Cut-off: 60 Median: 68 (26–93)	In the univariate analysis, an age of >60 years was associated with decreased survival (*p =* 0.04). No significance in the multivariate analysis
Sugitani et al. (2012) [36]	677	Surgery—45% EBRT—59% ChT—47%	Cut-off: 70 <70 (48%) ≥70 (52%)	An age of <70 years was an independent predictor for beneficial survival (HR = 1.28, 95% CI = 1.04–1.58, *p* = 0.020)
Haymart et al. (2013) [3]	2742	Surgery—50.2% RT—58.2% ChT—38.8%	≤44 (3.0%) 45–64 (27.5%) 65–74 (27.5%) 75–84 (30.4%) ≥85 (11.7%)	An age of ≥85 years was associated with greater mortality in the adjusted Cox regression model (HR = 3.43, 95% CI = 2.34–5.03, *p* < 0.05)
Dumke et al. (2014) [37]	40	Surgery—80% RT—98% ChT—15%	Median: 67 (38–84)	Age was not a significant prognostic factor in the uni- or multivariate analysis
Mohebati et al. (2014) [38]	83	Surgery alone—12% RT alone—4% ChT/RT—5% Surgery + RT + ChT—46%	Cut-off: 60 ≤60 (35%) >60 (65%)	1-year DSS (*p* = 0.012) in the univariate analysis ≤60 (52%) >60 (24%) No significance in the multivariate analysis
Polistena et al. (2014) [39]	79	Surgery—57% RT—59% ChT—100%	Cut-off: 75 <75 (53%) >75 (47%)	Patients <75 years and with tumors <5 cm in extent had the most favorable prognosis among subgroups in the univariate analysis (*p* < 0.05)
Sun et al. (2014) [12]	42	Surgery alone—29% EBRT alone—12% ChT alone—5% Surgery + RT—26% Surgery + RT/ChT—14% Surgery + ChT—10%	Cut-off: 55 <55 (33%) ≥55 (67%)	In the univariate analysis, an age of ≤55 years was significantly associated with improved 1- and 3-year overall survival rates (*p =* 0.012) No significance in the multivariate analysis
Ziveljevic et al. (2014) [17]	150	Surgery—57% Pre-OP RT—2.4% Post-OP RT—78.7% ChT—79%	≤50 (7.3%) 51–70 (73.3%) ≥70 (19.3%)	Younger age was an independent predictor of favorable survival (OR = 0.68, 95% CI = 0.49–0.95, *p =* 0.023)
Lo et al. (2015) [40]	15	Surgery—47% RT—20% ChT—0%	Median: 63 (36–73)	Age was not a significant prognostic factor in the uni- or multivariate analysis
Paunovic et al. (2015) [41]	150	Surgery—56.7% Pre-OP RT—2.4% Post-OP RT—78.8% ChT—2.4%	<40 (1.3%) 41–50 (6.1%) 51–60 (19.3%) 61–70 (54.0%) >70 (19.3%)	An age of <50 years is an independent predictor associated with overall survival (RR = 0.68, 95% CI = 0.49–0.95, *p* = 0.023)
Baek et al. (2016) [42]	329	RT/cCRT—15.2% Curative resection—28.6% Curative resection and adjuvant RT/cCRT—25.5% Curative resection and adjuvant ChT—3.0% ChT alone—3.0%	Cut-off: 70 <70 (51.7%) ≥70 (48.3%)	An age of ≥70 years was an independent predictor for poorer disease-specific survival (HR = 1.493, 95% CI = 1.134–1.965, *p* < 0.01)
Glaser et al. (2016) [43]	3552	Surgery—49.5% RT—58.7% ChT—41.6%	Cut-off: 65 <65 (31.6%) ≥65 (68.4%)	An age of <65 years was an independent predictor for improved overall survival (HR = 1.42, 95% CI = 1.26–1.60, *p* < 0.0005)
Käsmann et al. (2016) [44]	9	Surgery—78% RT—78% ChT—78%	Cut-off: 64 ≤64 (56%) >64 (44%)	Age was not a significant prognostic factor in the uni- or multivariate analysis
Lee et al. (2016) [13]	98 (ATC)	Surgery-based—58.2% EBRT-based—17.3% ChT—7.1%	Mean: 63.4 ± 13.4	Age at diagnosis in years achieved significance in the multivariate analysis (OR = 1.022, 95% CI = 0.01–1.10, *p* = 0.029) in a group, where resectability was adjusted with age, tumor size, WBC count and N status
Lennon et al. (2016) [45]	64	Surgery alone—17.2% RT alone—26.6% ChT alone—4.7% Surgery + RT—10.9% RT + ChT—9.4% Surgery + RT + ChT—12.5%	Cut-off: 70 Median: 72 (47–93)	In the univariate analysis, an age of >70 years was associated with improved overall survival (*p =* 0.041) No significance in the multivariate analysis
Liu et al. (2016) [6]	50	Total or extensive thyroidectomy—76% Palliative resection of cervical lymph nodes—6% RT—32% ChT—16%	Cut-off: 60 ≤60 (52%) >60 (48%)	Age was not a significant prognostic factor in the uni- or multivariate analysis
Pezzi et al. (2016) [5]	1288	Surgery (any neck, but only R2)—11.6% RT—47.7% ChT—53.8%	Cut-off: 65 Average: 70.4	An age of <65 years was an independent predictor for beneficial patient survival (HR = 1.317, 95% CI = 1.137–1.526, *p* < 0.001)
Wendler et al. (2016) [16]	100	Surgery—83% EBRT—81% ChT—56%	Cut-off: 70 <70 (46%) ≥70 (54%)	An age of <70 years was an independent predictor for beneficial survival (HR = 1.048, 95% CI = 1.015–1.082, *p* = 0.004)
Hvilsom et al. (2017) [46]	219	Thyroid surgery (R0—2)—50.7% Lymph node surgery—72% ChT/RT—Not reported	Median: 74 (30–94)	An age of ≤73.6 years was an independent predictor for improved overall survival (HR = 1.4, 95% CI = 1.0−2.0)
Jacobsen et al. (2017) [47]	31	Surgery—42% RT—100% ChT—74%	Median: 69 (26–87)	In the univariate analysis, age at diagnosis in years achieved significance (HR = 1.02, 95% CI = 0.98−1.07) No significance in the multivariate analysis
Park et al. (2018) [48]	41	Surgery + RT + ChT—39% Surgery + RT—12.2% RT + ChT—36.6% RT alone—12.2%	Cut-off: 65 <65 (31.7%) ≥65 (68.3%)	Age was not associated with better/poorer outcome in the univariate analysis (HR = 1.44, 95% CI = 0.69–3.01, *p* = 0.328)
Takahashi et al. (2018) [49]	33	Surgery—39% ChT—52% CRT—45%	Median 68 (41–87)	Age (≥ median vs. < median) was not associated with better/poorer outcome in the univariate analysis (HR= 1.22, 95% CI = 0.57–2.60, *p* = 0.605)
Corrigan et al. (2019) [15]	28	Surgery—71.4% EBRT—75% ChT—50%	Not reported	Younger age is an independent predictor for better overall survival (HR = 1.079; 95% CI = 1.022−1.139; *p =* 0.006)
Fan et al. (2019) [22]	104	ChT/RT—95.2% Surgery + RT + ChT— 51%	Cut-off: 70 Median: 63.5 (28–87)	In the univariate analysis, the age of <70 years was significantly associated with improved overall survival (*p =* < 0.001) No significance in the multivariate analysis
Huang et al. (2019) [50]	735	Surgery—26% RT—36% ChT—31% No treatment—22%	Cut-off: 70 Median: 70 IQR: 60–80	Age at diagnosis in years achieved significance in the multivariate analysis (HR = 1.022, 95% CI = 1.010–1.034, *p* < 0.001) No difference in favor for the subgroups ≤/>70 years in terms of total thyroidectomy
Li et al. (2019) [51]	1048	Primary surgery—45% EBRT—55% ChT—42%	Cut-off: 65 <65 (33%) ≥65 (67%)	An age of ≥65 years was an independent predictor for overall survival (HR = 1.34, 95% CI = 1.16–1.55, *p* < 0.001)
De Ridder et al. (2020) [52]	812	Surgery—12% Surgery + RT—15% Surgery + cCRT—2% Surgery + RT + ChT—3% Surgery + ChT—1% RT—28% cCRT—1% RT + ChT—3% ChT—1%	Median: 73 (29–99)	Age at diagnosis was an independent prognostic factor for poorer outcome (HR = 1.014, 95% CI = 1.006–1.020, *p* < 0.001)
Gui et al. (2020) [53]	1404	Surgery—44% EBRT—59% ChT—not reported	Cut-off: 65 <65 (34%) ≥65 (66%)	An age of ≥65 years was an independent predictor for worse overall survival (HR = 1.525, 95% CI = 1.326–1.752, *p* < 0.001)
Lin et al. (2020) [54]	1567/717	Surgery—566/1567 (36%) Not reported for RT/ChT	Median: 71 (23–100)	Younger age is an independent predictor for overall survival (HR = 1.02, 95% CI = 1.01–1.02, *p* < 0.001)
Saeed et al. (2020) [55]	496	Surgery—100% Adjuvant EBRT—76% Adjuvant Chemotherapy—59% Adjuvant CRT—56.4%	Cut-off: 65 <65 (42%) ≥65 (58%)	In the univariate analysis, an age of ≥65 years was a significant prognostic factor for overall survival (*p =* 0.04) No significance in the multivariate analysis

External Beam Radiation Therapy (EBRT), Chemotherapy (ChT), Overall Survival (OS), Hazard Ratio (HR), Odds Ratio (OR), Relative Risk (RR), Radiation Therapy (RT), Confidence Interval (CI), Chemoradiotherapy (CRT), concurrent chemoradiotherapy (cCRT), Disease Specific Survival (DSS).

**Table 2:** Patient and treatment characteristics of the pooled patient cohort.

Parameter	Value (%)
Total	186 (100)
Age, years (range)	68 (35–92)
Gender	
Male	54 (39)
Female	60 (44)
Unknown	24 (17)
UICC stage	
IVA/B	113 (61)
IVC	51 (27)
Unknown	22 (12)
Surgery	
No	91 (49)
Yes	95 (51)
Radiotherapy	
No	34 (18)
Yes	152 (82)
Sequential or concurrent chemotherapy	
No	72 (39)
Yes	114 (61)
Multimodal treatment	
No	112 (60)
Yes	74 (40)

Union of International Cancer Control (UICC).

**Table 3:** Patient and treatment characteristics of the propensity score cohort.

Parameter	Entire PSM Cohort,	Subgroup with	Subgroup with	*p*-Value
	*N* (%)	Patients Aged < 65 Years, *N* (%)	Patients Aged ≥ 65 Years, *N* (%)	
Total	138 (100)	69 (50)	69 (50)	
Age, years (range)	65 (35–92)	56 (35–64)	74 (65–92)	<0.001
Gender				
Male	54 (39)	33 (48)	21 (30)	
Female	60 (44)	22 (32)	38 (55)	0.009
Unknown	24 (17)	14 (20)	10 (15)	
UICC stage				
IVA/B	92 (67)	46 (67)	46 (67)	0.999
IVC	46 (33)	23 (33)	23 (33)	
Surgery				
No	59 (43)	27 (39)	32 (46)	0.391
Yes	79 (57)	42 (61)	37 (54)	
Radiotherapy				
No	14 (10)	8 (12)	6 (9)	0.574
Yes	124 (90)	61 (88)	63 (91)	
Sequential or concurrent chemotherapy				
No	54 (39)	18 (26)	36 (52)	0.002
Yes	84 (61)	51 (74)	33 (48)	

Union for International Cancer Control (UICC).

**Table 4:** Eighth edition of the Union for International Cancer Control Tumor–Node–Metastasis (UICC TNM) classification.

Stage	Eighth Edition of UICC TNM
IVA	T1–3a, N0 and M0 T1: Tumor ≤ 2 cm in the greatest dimension limited to the thyroid T2: Tumor > 2 cm but ≤4 cm in the greatest dimension limited to the thyroid T3a: Tumor > 4 cm limited to the thyroid
IVB	T1–3a, N1 and M0 or T3b–T4b, any N and M0 T3b: Gross extrathyroidal extension invading only strap muscles (sternohyoid, sternothyroid, thyrohyoid and omohyoid muscles) from a tumor of any size T4a: Gross extrathyroidal extension invading subcutaneous soft tissues, larynx, trachea, esophagus or recurrent laryngeal nerve from a tumor of any size T4b: Gross extrathyroidal extension invading prevertebral fascia or encasing a carotid artery or mediastinal vessels from a tumor of any size
IVC	Any T, any N and M1

Union of International Cancer Control (UICC), Tumor (T), Node (N), Metastases (M).

**Table 5:** Patient- and treatment-related characteristics.

Parameter	*n*
Age, years	
<74	11 (42%)
≥74	15 (58%)
Gender	
Male	13 (50%)
Female	13 (50%)
KPS, %	
≤70	12 (46%)
>70	14 (54%)
T stage	
2–3	2 (8%)
4	24 (92%)
N stage	
0	10 (39%)
1	16 (62%)
M stage	
0	10 (39%)
1	16 (62%)
Number of metastatic sites	
1	7 (44%)
2	7 (44%)
3	1 (6%)
4	1 (6%)
UICC stage	
IVA	1 (4%)
IVB	9 (35%)
IVC	16 (62%)
Surgery	
No	14 (54%)
Yes	12 (46%)
Chemotherapy	
No	13 (50%)
Yes	13 (50%)
Treatment	
RT/CRT	14 (54%)
S+CRT	12 (46%)
Resection status	
R0	1 (8%)
R1	7 (58%)
R2	4 (33%)
EQD2 level	
≤49	14 (54%)
>49	12 (46%)
RT technique	
3D-CRT	17 (65%)
IMRT	9 (35%)

Karnofsky performance status (KPS), Tumor (T), Node (N), Metastases (M), Union of International Cancer Control (UICC), Radiation Therapy (RT), Chemoradiotherapy (CRT), three-dimensional conformal radiotherapy (3D-CRT), equivalent dose in 2 Gy fractions (EQD2), intensity-modulated radiation therapy (IMRT).

**Table 6:** Uni- and multivariate analysis of overall survival (OS).

	Univariate Analysis				Multivariate Analysis			
Parameter	At 6 Months	At 12 Months	At 24 Months	*p*-Value	*p*-Value	Hazard Ratio	95% CI Lower	95% CI Upper
Age, years					-	-	-	-
≤74	55%	36%	12%	0.15				
>74	22%	10%	10%					
Gender					-	-	-	-
Male	23%	23%	12%	0.45				
Female	46%	19%	9%					
KPS, %								
≤70	0%	0%	0%	<0.001	0.357	0.422	0.068	2.64
>70	64%	40%	20%					
N stage					-	-	-	-
0	50%	40%	27%	0.028				
1	25%	8%	0%					
M stage					-	-	-	-
0	70%	47%	31%	0.001				
1	13%	6%	0%					
UICC stage								
IVA	100%	100%	100%	0.004	0.618	1.449	0.337	6.223
IVB	67%	40%	27%					
IVC	13%	6%	0%					
Surgery					-	-	-	-
No	7%	0%	0%	<0.001				
Yes	67%	42%	21%					
Chemotherapy					-	-	-	-
No	31%	21%	21%	0.78				
Yes	39%	23%	0%					
Treatment								
RT/CRT	7%	0%	0%	<0.001	0.524	0.519	0.069	3.911
S+CRT	67%	42%	21%					
EQD2 level								
≤49	7%	0%	0%	<0.001	0.426	0.562	0.136	2.32
>49	67%	42%	24%					
RT technique					-	-	-	-
3D-CRT	29%	12%	6%	0.18				
IMRT	44%	44%	22%					

Karnofsky performance status (KPS), Node (N), Metastases (M), Union of International Cancer Control (UICC), Radiation Therapy (RT), Chemoradiotherapy (CRT), three-dimensional conformal radiotherapy (3D-CRT), equivalent dose in 2 Gy fractions (EQD2), intensity-modulated radiation therapy (IMRT).

**Table 7:** Uni- and multivariate analysis of progression-free survival (PFS).

	Univariate Analysis				Multivariate Analysis			
Parameter	At 3 Months	At 6 Months	At 12 Months	*p*-Value	*p*-Value	Hazard Ratio	95% CI Lower	95% CI Upper
Age, years					-	-	-	-
≤74	27%	27%	27%	0.29				
>74	27%	7%	7%					
Gender					-	-	-	-
Male	31%	23%	23%	0.82				
Female	23%	8%	8%					
KPS, %								
≤70	17%	0%	0%	0.025	0.532	1.625	0.354	7.452
>70	36%	29%	29%					
N stage								
0	60%	40%	40%	<0.001	0.325	1.812	0.555	5.919
1	6%	0%	0%					
M stage								
0	40%	30%	30%	0.03	0.373	1.939	0.452	8.318
1	19%	6%	6%					
UICC stage					-	-	-	-
IVA	100%	100%	100%	0.056				
IVB	33%	22%	22%					
IVC	19%	6%	6%					
Surgery					-	-	-	-
No	7%	0%	0%	<0.001				
Yes	50%	33%	33%					
Chemotherapy					-	-	-	-
No	15%	15%	15%	0.36				
Yes	39%	15%	15%					
Treatment								
RT/CRT	7%	0%	0%	<0.001	0.352	0.405	0.06	2.718
S+CRT	50%	33%	33%					
EQD2 level								
≤49	14%	0%	0%	0.006	0.944	0.947	0.207	4.34
>49	42%	33%	33%					
RT technique					-	-	-	-
3D-CRT	18%	6%	6%	0.18				
IMRT	44%	33%	33%					

Karnofsky performance status (KPS), Tumor (T), Node (N), Metastases (M), Union of International Cancer Control (UICC), Radiation Therapy (RT), Chemoradiotherapy (CRT), three-dimensional conformal radiotherapy (3D-CRT), equivalent dose in 2 Gy fractions (EQD2), intensity-modulated radiation therapy (IMRT).
